# IL-4 and IL-13 induce equivalent expression of traditional M2 markers and modulation of reactive oxygen species in human macrophages

**DOI:** 10.1038/s41598-023-46237-2

**Published:** 2023-11-10

**Authors:** Tara E. Scott, Caitlin V. Lewis, Mingyu Zhu, Chao Wang, Chrishan S. Samuel, Grant R. Drummond, Barbara K. Kemp-Harper

**Affiliations:** 1 Cardiovascular Disease Program, Department of Pharmacology, Biomedicine Discovery Institute, Monash University, Clayton, VIC Australia; 2 Department of Physiology, Anatomy and Microbiology, School of Life Sciences, La Trobe University, Melbourne, VIC Australia

**Keywords:** Diseases, Cell signalling, Cytokines, Monocytes and macrophages

## Abstract

In cardiovascular disease, pathological and protective roles are reported for the Th2 cytokines IL-4 and IL-13, respectively. We hypothesised that differential effects on macrophage function are responsible. Type I and II receptor subunit (IL-2Rγ, IL-4Rα and IL-13Rα1) and M2 marker (MRC-1, CCL18, CCL22) expression was assessed via RT-qPCR in IL-4- and IL-13-treated human primary macrophages. Downstream signalling was evaluated via STAT1, STAT3 and STAT6 inhibitors, and IL-4- and IL-13-induced reactive oxygen species (ROS) generation assessed. IL-4 and IL-13 exhibited equivalent potency and efficacy for M2 marker induction, which was attenuated by STAT3 inhibition. Both cytokines enhanced PDBu-stimulated superoxide generation however this effect was 17% greater with IL-4 treatment. Type I IL-4 receptor expression was increased on M1-like macrophages but did not lead to a differing ability of these cytokines to modulate M1-like macrophage superoxide production. Overall, this study did not identify major differences in the ability of IL-4 and IL-13 to modulate macrophage function, suggesting that the opposing roles of these cytokines in cardiovascular disease are likely to be via actions on other cell types. Future studies should directly compare IL-4 and IL-13 in vivo to more thoroughly investigate the therapeutic validity of selective targeting of these cytokines.

## Introduction

The Th2 cytokines, interleukin-4 (IL-4) and interleukin-13 (IL-13), are well recognised for their roles in allergic diseases, such as asthma. In addition, they exhibit anti-inflammatory properties, inhibiting type I inflammation, i.e., interferon-γ (IFN-γ)-induced, and promoting polarisation of macrophages towards the reparative, M2-like (herein referred to as M2) phenotype^1,2^. This alternative activation of macrophages by Th2 cytokines is of particular interest in cardiovascular diseases, such as atherosclerosis, hypertension, and myocardial infarction (MI) where M2 macrophages promote atherosclerotic plaque stabilisation and reparative connective tissue formation^3,4^. Interestingly, although IL-4 and IL-13 share many similar biological activities and promote M2 polarisation, opposing roles of these Th2 cytokines have been reported in cardiovascular pathologies.

This is evident in the setting of atherosclerosis where a pro-atherogenic role of IL-4 has been reported^4^. Moreover, deficiency in IL-4 inhibits atherogenesis^3^. In addition, IL-4 can negatively impact macrophage function^5–7^ in vascular pathologies. Furthermore, IL-4 plays a detrimental pro-fibrotic role in hypertension-induced cardiac remodelling and dysfunction in mice^8,9^.

In contrast, it has been reported that IL-4 has no effect on atherosclerotic lesion development^10^ and conversely, that IL-4 exhibits atheroprotective effects^11^. Moreover, one study suggested that exogenous IL-4 may serve as a potential treatment for myocardial infarction (MI) by promoting reparative connective tissue formation in the infarct area, but not pathological interstitial fibrosis, potentially via an impact on macrophage function^12,13^. Nevertheless, a significant body of evidence still suggests that IL-4 may have detrimental effects in cardiovascular disease^3–9^.

Interestingly, despite being closely related to IL-4, cardioprotective actions of IL-13 have been demonstrated^14^. Furthermore, IL-13 is increased in human asymptomatic plaques, but a similar correlation does not exist for IL-4^15^. In addition, increased intima-media thickness (a surrogate for subclinical atherosclerosis) is associated with lower circulating IL-13^16^. Likewise, IL-13 expression is elevated in the left and right ventricular myocardium of mice following MI^17^. Taken together, these findings suggest that despite both promoting a M2 phenotype, IL-4 and IL-13 may play opposing roles in cardiovascular disease. The mechanisms underlying these opposing actions, however, remain unknown but may reflect differential effects of these cytokines on M2 macrophage function.

There are two receptor complexes that IL-4 and IL-13 act upon, the type I and type II IL-4 receptor. The type I receptor is comprised of the IL-2Rγ and IL-4Rα subunits, that are only activated by IL-4. By contrast, both IL-4 and IL-13 can target the type II receptor which shares the IL-4Rα subunit and consists of the additional IL-13Rα1 chain^18^. Of note, IL-13 also exhibits high affinity for a ‘decoy’ cell surface receptor IL-13Rα2^19^, however, its constitutive expression in macrophages has not been reported. Macrophages are one of just a few cell types expressing both the type I and type II IL-4 receptor^18,20^ and thus IL-4 has an additional target on macrophages, as compared to IL-13. Recent evidence has demonstrated a beneficial role for both components of the type II receptor, (i.e., a potential IL-13 bias) in a cardiovascular setting.

As well as displaying differences in receptor binding and subunit assembly, IL-4 and IL-13 also utilise different downstream signalling mechanisms. When IL-4 binds the type I receptor, janus-activated kinase (JAK) 1 linked to the IL-4Rα subunit is activated and subsequent phosphorylation of signal transducer and activator of transcription (STAT)3 and STAT6 takes place^21^.

Activation of the type II receptor by IL-4 leads to activation of the JAK2/STAT3 pathway (IL-4Rα) or tyrosine kinase 2 (TYK2)/STAT6 pathway linked to the IL-13Rα1 subunit^21^, with activation of the type II receptor by IL-13 but not IL-4 also activating the TYK2/STAT1 cascade. The many shared actions of IL-4 and IL-13 can likely be attributed to their overlapping use of receptor subunits and activation of various JAKs leading to the convergence of both receptors on the STAT3 and STAT6 pathways^21^. Not surprisingly then, STAT6 is known to regulate the expression of a number of M2 markers such as mannose receptor (MRC-1), Arginase 1 (Arg1) and IL-4Rα^22,23^. More importantly though, potential differences in the actions of the cytokines IL-4 and IL-13 may be due to type II receptor activation by IL-13, but not IL-4, recruiting STAT1 signalling^21^.

An ability of IL-4 and IL-13 to target distinct receptors and modulate the production of effector molecules such as reactive oxygen species (ROS) may contribute to their contrasting actions. ROS production is elevated in many cardiovascular diseases^24,25^ and plays a key role in the development of atherosclerosis, hypertension and MI, contributing to tissue damage, inflammation and endothelial dysfunction^26,27^. Although ROS production has generally been regarded as a function of M1 macrophages, M2 macrophages also express the superoxide generating enzymes, NADPH oxidases, and thus have the capacity to generate ROS. Whilst IL-4 has been shown to target endothelial cells to promote vascular oxidative stress^28^, its impact on M2 macrophage-derived ROS generation has not been thoroughly studied. With the capacity to activate both the type I and type II IL-4 receptor, leading to downstream signalling associated with enhanced NADPH oxidase activity^29,30^, IL-4 may serve as a greater stimulus for macrophage ROS generation than IL-13 in the setting of cardiovascular disease.

Given the central inflammatory role that macrophages play in cardiovascular disease, we hypothesised that the opposing effects of IL-4 and IL-13 may be via their differential modulation of M2 macrophage function, specifically the release of ROS. This study aimed to compare the ability of IL-4 and IL-13 to promote M2 polarisation and ROS generation in human primary macrophages. Moreover, given we observed enhanced expression of the type I IL-4 receptor on M1 macrophages, a potential difference in the ability of these Th2 cytokines to modulate M1 function was also investigated. The present study has the potential to inform on more selective targeting of IL-4 and IL-13 and their downstream effector molecules in cardiovascular disease.

## Materials and methods

### Monocyte isolation

Primary human monocytes were isolated from healthy human blood donor buffy coats obtained from the Australian Red Cross Blood Service (Melbourne, Australia) in accordance with Material Supply Agreement 16-05VIC-11. Buffy coats were mixed with phosphate buffered saline (PBS; without Ca^2+^or Mg^2+^; Sigma-Aldrich), supplemented with 0.5% fetal bovine serum (FBS; Sigma-Aldrich) and 2 mM ethylenediaminetetraacetic acid (EDTA; Sigma-Aldrich) and layered onto Ficoll-Paque PLUS (GE Healthcare no. 17-144) for density gradient centrifugation (400*g*, 40 min, acceleration = 1, deceleration = 0). The peripheral blood mononuclear cell (PBMC) layer was collected and monocytes isolated using a human pan monocyte isolation kit (Miltenyi Biotec no. 130-096-537), according to the manufacturer’s instructions. The purity of the monocyte population was confirmed to be at least 85% as determined by Flow cytometry using CD14^+^/CD16^+^ expression^31^.

### Monocyte to macrophage differentiation and macrophage polarisation

Isolated donor blood-derived primary monocytes were seeded into 6-well plates (2 × 10^6^ cells/well; RNA and protein extraction) or 96-well plates (2 × 10^5^ cells/well; L-012-enhanced chemiluminescence) and maintained in a humidified incubator (Sanyo MCO-18AIC CO_2_ incubator, Quantum Scientific, USA) at 37 °C with 5% CO_2_. Monocytes were differentiated to macrophages by culturing for 7 days in RPMI 1640 Glutamax medium (Gibco Life Technologies), supplemented with 10% FBS, 1 × antibiotic/antimycotic (Gibco Life Technologies, USA), 1 mM sodium pyruvate (Sigma-Aldrich), 1 × non-essential amino acids (NEAA; Gibco Life Technologies) and 50 ng/ml macrophage colony stimulating factor (M-CSF; Miltenyi Biotec no. 130-096-491). Following 7 day macrophage differentiation, culture media was replaced without M-CSF and macrophages were either left untreated (MΦ), treated with 20 ng/ml IFN-γ (Sigma-Aldrich no. I3265) and 100 ng/ml lipopolysaccharide (LPS; Sigma-Aldrich no. L2630, *E. coli* 0111:B4 strain) for M1 polarisation, or with either interleukin-4 (IL-4; Sigma-Aldrich no. I4269) or interleukin-13 (IL-13; Sigma-Aldrich no. I1771) at concentrations ranging from 0.005 to 50 ng/ml for 24 h. In a separate set of experiments, M1-polarised macrophages (20 ng/ml IFN-γ and 100 ng/ml LPS, 18 h) were then treated with 50 ng/ml IL-4 or IL-13 for 6 h and superoxide generation detected via L-012 chemiluminescence^31^.

To investigate the signalling pathways utilised by IL-4 and IL-13; a number of signal transduction inhibitors were employed. Fludarabine 100 μM (STAT1 inhibitor; Selleckchem, USA), Stattic 10 μM (STAT3 inhibitor; Selleckchem, USA), and AS1517499 100 nM (STAT6 inhibitor; Axon MedChem, Netherlands). All inhibitors were added 30 min prior to IL-4 or IL-13 treatment (both 2.5 ng/ml, 24 h), and remained present for the 24-h duration of treatment.

### RNA extraction and real time-qPCR

Total RNA was extracted from macrophages using the RNeasy Mini Kit (Qiagen) according to the manufacturer’s instructions. RNase-free DNase (Qiagen) was used to remove any contaminating DNA. The amount of RNA in each sample was quantified using the Nanodrop 1000D spectrophotometer (ThermoScientific), which measures absorbance at 260 nm and 280 nm. An A_260_:A_280_ ratio of 2 or more was considered sufficiently pure. 0.5 µg of RNA from each sample was reverse transcribed into cDNA using the High Capacity cDNA Reverse Transcription Kit (Applied Biosystems) with the reaction run in a thermal cycler (BioRad MyCycler, BioRad Laboratories). The resultant cDNA was used as a template for real time PCR with predesigned Taqman^®^ primers and probes for IL-2Rγ, IL-4Rα, IL-13Rα1, MRC-1, CCL18, CCL22, CYBB (NOX2), NCF1 (p47phox), NCF2 (p67phox), CYBA (p22phox), NOX1, NOX4, NOX5 and 18S (Applied Biosystems) was used as a housekeeping gene. Real-time PCR was run in triplicate on the CFX96 Touch™ Real-Time PCR Detection Machine (BioRad Laboratories). Gene expression was normalised to the housekeeping gene 18S and expressed relative to the average MΦ value using the comparative cycle threshold (Ct) method with the formula: fold change = 2^−ΔΔCt^^31,32^.

### Protein extraction and Western blotting

Total protein from macrophage lysates was collected in 1.5 × Laemmli buffer (7.5% glycerol; 3.75% β-mercaptoethanol; 2.25% sodium dodecyl sulfate (SDS); 75 mM Tris–HCl pH 6.8; 0.004% bromophenol blue), cell debris was cleared by centrifugation (13,000 rpm, 10 min, 4 °C) and supernatants were collected. Protein concentrations were determined using a modified Lowry protocol (RCDC colorimetric protein assay kit; BioRad Laboratories). 20 µg of protein in 1.5 × Laemmli buffer was loaded into 7.5% or 10% polyacrylamide gels and proteins were separated by SDS-PAGE and transferred onto low fluorescence polyvinylidene fluoride (LF PVDF) membranes using the Bio-Rad Trans Blot Turbo transfer system (Bio-Rad Laboratories). Membranes were blocked with 5% skim milk in Tris-Buffered Saline (TBS; 200 mM Tris, 150 mM NaCl, pH 7.5) with 0.1% tween-20 for 1 h and subsequently probed with primary antibodies against IL-2Rγ (1:500; Abcam no. ab180698), IL-4Rα (1:500; Abcam no. ab131058), IL-13Rα1 (1:200; Abcam no. ab140367), NOX2 [1:500; Santa-Cruz no. sc-130549 (CL5)], p47phox (1: 1000; BD Transduction Laboratories no. 610354), p67phox (1:2000; EMD Millipore no. 07-002), and GAPDH (1: 20,000; Abcam no. ab8245) overnight at 4 °C. 1 h incubation with horseradish peroxidase (HRP)-conjugated anti-rabbit (1:10,000; Dako) or anti-mouse (1:10,000; Jackson ImmunoResearch Laboratories) secondary antibodies was then performed and protein bands visualised using Clarity ECL substrate (BioRad Laboratories) and the ChemiDoc MP system (BioRad Laboratories). Densitometries of protein bands were quantified using Image Lab Software (BioRad Laboratories) and normalised to the housekeeping protein GAPDH. Fold change in protein expression was expressed relative to the average MΦ value^31^.

### Superoxide detection via L-012-enhanced chemiluminescence

Primary macrophages were seeded and treated on white 96-well tissue culture plates (Perkin Elmer) at 2 × 10^5^ cells/well. Groups were set up in quintuplicate with a cell free control group, comprising media alone, included to provide a background reference. On the day of experimentation, culture medium was removed and cells were washed and incubated in warmed Krebs-HEPES buffer (in mM: NaCl 118; KCl 4.7; KH_2_PO_4_ 1.2; MgSO_4_·7H_2_O 1.2; CaCl_2_ 2.5; NaHCO_3_ 25; glucose 11.7; HEPES 20, pH 7.4) and background chemiluminescence measured for 30 min. Chemiluminescence was measured using a Chameleon Luminescence Plate Reader (Hidex Ltd, Turku, Finland) and data acquired using the MicroWin (Mikrotek, Overath, Germany) data acquisition system. 100 μM L-012 (Wako Pure Chemical Industries) was then added to each well and basal superoxide levels were monitored for 30 min. Finally, the protein kinase C (PKC) activator, phorbol 12,13-dibutyrate (PDBu, 10 μM) was added to each well and superoxide production was then measured for a further 60 min. Peak PDBu-stimulated superoxide production was quantified as the average of 5 cycles at the peak of the signal for each group with the basal signal (average of the final 5 basal readings) subtracted^31^.

### Statistical analysis

All data are expressed as mean ± SEM with n = 4–8. Comparisons of multiple treatment groups were made using an ordinary one-way analysis of variance (ANOVA) with a Dunnett’s (comparison vs MΦ), Sidak’s (selected comparisons) or Tukey’s (comparison between all groups) post hoc test. For L-012 chemiluminescence experiments, comparisons were made using a repeated measures one-way ANOVA with Sidak’s or Tukey’s post hoc tests. Concentration response curves to IL-4 and IL-13 were fitted to a sigmoidal logistic equation and EC_50_ values determined based on fitted curves for the group data. P < 0.05 was considered to be statistically significant and data were graphed and analysed using GraphPad Prism 9.2 software.

## Results

### Human primary macrophages express equivalent levels of type I and type II IL-4 receptors

**Figure 1:**
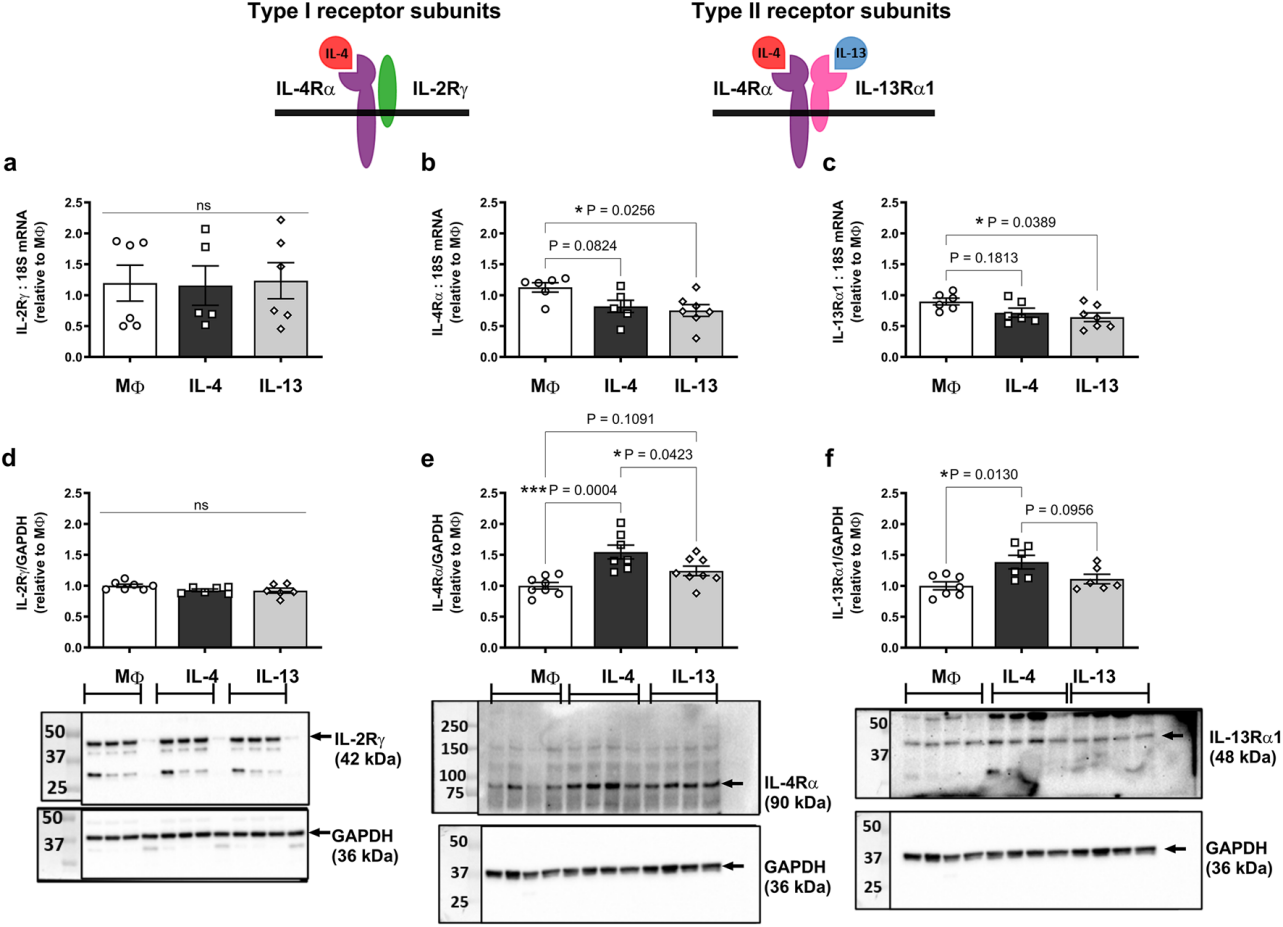
Type I and II IL-4 receptor subunits are expressed in human primary macrophages treated with IL-4 and IL-13. M-CSF-differentiated human primary macrophages were left untreated (MΦ) or treated with 50 ng/ml IL-4 or IL-13 for 24 h. mRNA levels of (**a**) IL-2Rγ, (**b**) IL-4Rα and (**c**) IL-13Rα1 were determined by RT-qPCR and expressed relative to the average MΦ value, n = 5–7. Protein levels of (**d**) IL-2Rγ, (**e**) IL-4Rα and (**f**) IL-13Rα1 were determined by western blotting and expressed relative to the average MΦ value, n = 6–8. Representative blots depicting n = 3–4 shown beneath each graph with GAPDH used as a loading control. Blots have been cut to probe for the protein of interest and GAPDH. Original blots are presented in Supplementary Fig. 2. The structure of the type I and II IL-4 receptor complexes are shown above data. Results presented as mean ± SEM and expressed relative to MΦ (one-way ANOVA followed by Tukey’s post hoc test).

To investigate the relative expression of type I and type II receptors in human primary macrophages, mRNA expression was first assessed for the type I subunit (IL-2Rγ), the shared IL-4 receptor subunit (IL-4Rα), the type II subunit (IL-13Rα1), and the decoy receptor, (IL-13Rα2). RT-qPCR analysis revealed very similar mRNA expression levels of the type I and type II receptor subunits in untreated (MΦ) M-CSF-differentiated macrophages (Ct values = 25–27) and, as expected, no detectable expression of IL-13Rα2 (Table 1). We then sought to elucidate whether IL-4 and IL-13 treatment (50 ng/ml; 24 h) altered receptor expression. No change in expression of the type I receptor subunit, IL-2Rγ was detected at either the mRNA or protein level (Fig. 1a,d).

**Table 1:** mRNA expression of type I and II IL-4 receptor subunits in untreated (MΦ) human primary macrophages.

Type I and II IL-4 receptor subunit	Ct (cycle threshold)
IL-2Rγ (type I)	27.3 ± 0.3 (moderate)
IL-4Rα (type I & II)	26.1 ± 0.4 (moderate)
IL-13Rα1 (type II)	25.8 ± 0.3 (moderate)
IL-13Rα2 (decoy)	Not detected

Cts determined using RT-qPCR with equivalent cDNA loading for each gene, n = 7, mean ± SEM.

Whilst IL-13 treatment decreased both IL-4Rα and IL-13Rα1 mRNA expression (*P = 0.03 and *P = 0.04, respectively), IL-4 caused no significant change in expression of either subunit although a trend for the same effects were observed (P = 0.08 and P = 0.18, respectively; Fig. 1b,c). This effect was not reflected at the level of the protein, with a significant 1.5-fold increase in expression of both subunits in response to IL-4 (***P = 0.0004 for IL-4Rα, and *P = 0.01 for IL-13Rα1) but no significant change following IL-13 treatment (Fig. 1e,f).

### IL-4 and IL-13 upregulate M2 marker expression in human primary macrophages with equivalent potency and efficacy

**Figure 2:**
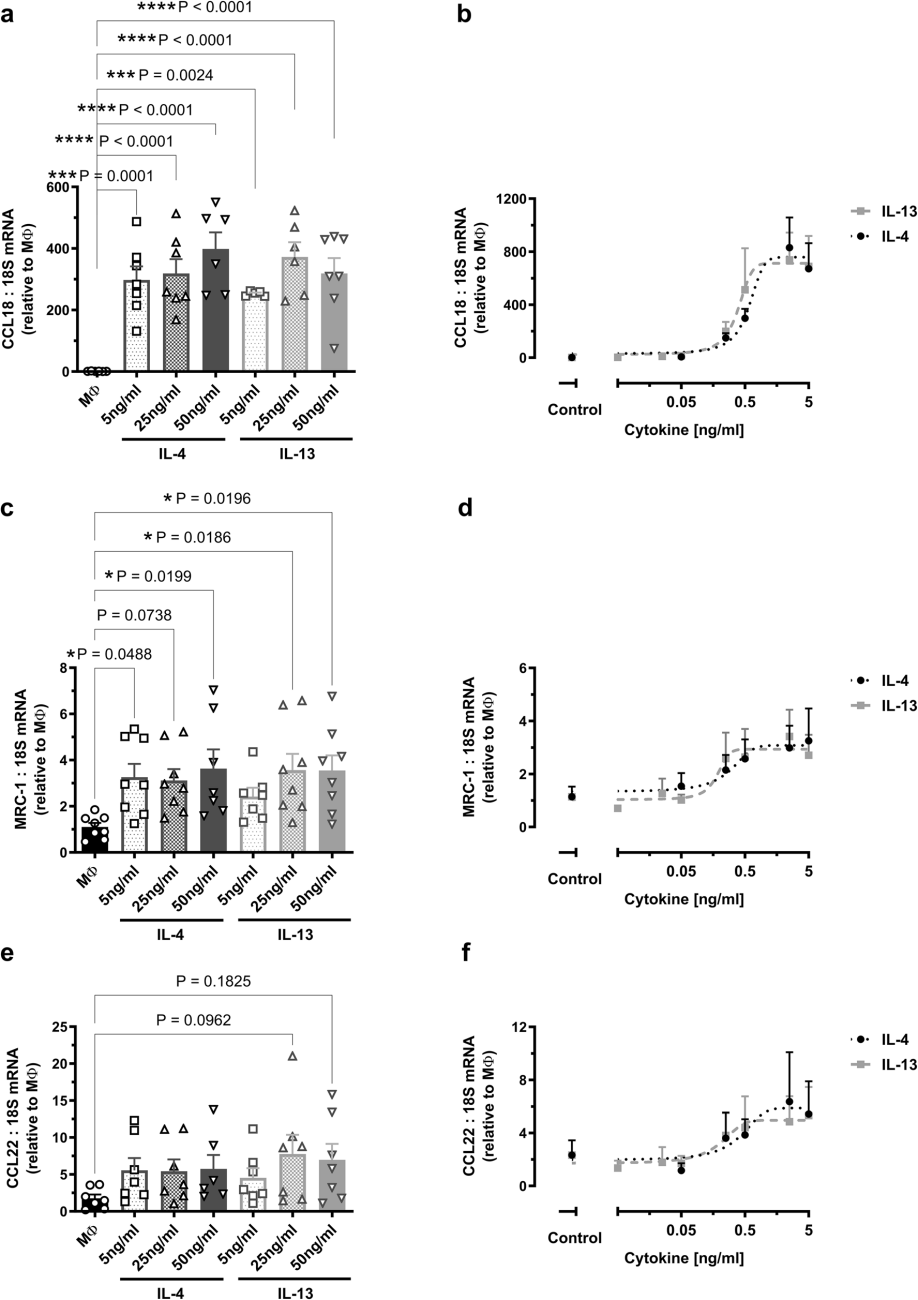
Potency and efficacy of IL-4 and IL-13 induction of M2 marker expression in human primary macrophages. M-CSF-differentiated human primary macrophages were left untreated (MΦ) or treated with either IL-4 or IL-13 for 24 h. Left panel: Fold changes in mRNA expression of (**a**) CCL18, (**c**) MRC-1 and (**e**) CCL22 treated with 5, 25 or 50 ng/ml IL-4 or IL-13, n = 6–8. Right panel: untreated (control) and concentration-dependent fold-changes in mRNA expression of (**b**) CCL18, (**d**) MRC-1, and (**f**) CCL22 following treatment with IL-4 or IL-13 (0.005–5 ng/ml, 24 h), n = 6–7. Results presented as mean ± SEM and expressed relative to the average MΦ value (one-way ANOVA followed by Dunnett’s post hoc test).

Despite increased IL-4Rα protein expression in IL-4-treated cells, and the potential for signalling through both receptor complexes by IL-4, no differences in the induction of M2 marker expression were observed between IL-4 and IL-13. Treatment of M-CSF-differentiated human primary macrophages with 5–50 ng/ml of IL-4 or IL-13 for 24 h, led to similarly increased expression of all three of the M2 markers examined. The greatest impact observed was for Chemokine C–C motif ligand 18 (CCL18) with mRNA expression increased by 300–400-fold (all concentrations P ≤ 0.002; Fig. 2a), followed by a ~ 3 fold elevation for mannose receptor C type-1 (MRC-1/CD206; majority of  concentrations P ≤ 0.05; Fig. 2c) and a trend towards an increase for CCL22 (Fig. 2e) which did not reach statistical significance (P = 0.1 and P = 0.18 for IL-13 25 ng/ml and 50 ng/ml, respectively).

As the effects of IL-4 and IL-13 were not concentration-dependent over the range 5–50 ng/ml, further experiments were performed using lower concentrations of the cytokines (0.005–5 ng/ml, Fig. 2b,d,f) to provide a measure of potency. IL-4 and IL-13 increased the expression of all three M2 macrophage markers with a similar potency and efficacy as demonstrated by EC_50_ and maximal responses (Table 2).

**Table 2:** Potency and efficacy of IL-4 and IL-13 induction of M2 marker mRNA expression in human primary macrophages.

	IL-4		IL-13	
	EC_50_ (ng/ml)	Maximum (fold-change relative to MΦ)	EC_50_ (ng/ml)	Maximum (fold-change relative to MΦ)
CCL18	0.57 ± 0.15	758 ± 86.6	0.38 ± 0.1	712 ± 108
MRC-1	0.26 ± 0.19	3.1 ± 0.5	0.18 ± 0.13	2.9 ± 0.42
CCL22	0.35 ± 0.31	5.9 ± 1.4	0.17 ± 0.15	5.0 ± 1.0

EC_50_ values determined based on the fitted curve for group data, n = 6–7, mean ± SEM.

### STAT3, but not STAT1 or STAT6 inhibition, attenuates IL-4- and IL-13-induced expression of M2 macrophage markers

To elucidate and compare the mechanism(s) by which IL-4 and IL-13 promote M2 macrophage marker expression, a variety of inhibitors of downstream signal transduction pathways known to be associated with IL-4 and IL-13 receptor signalling were studied.

**Figure 3:**
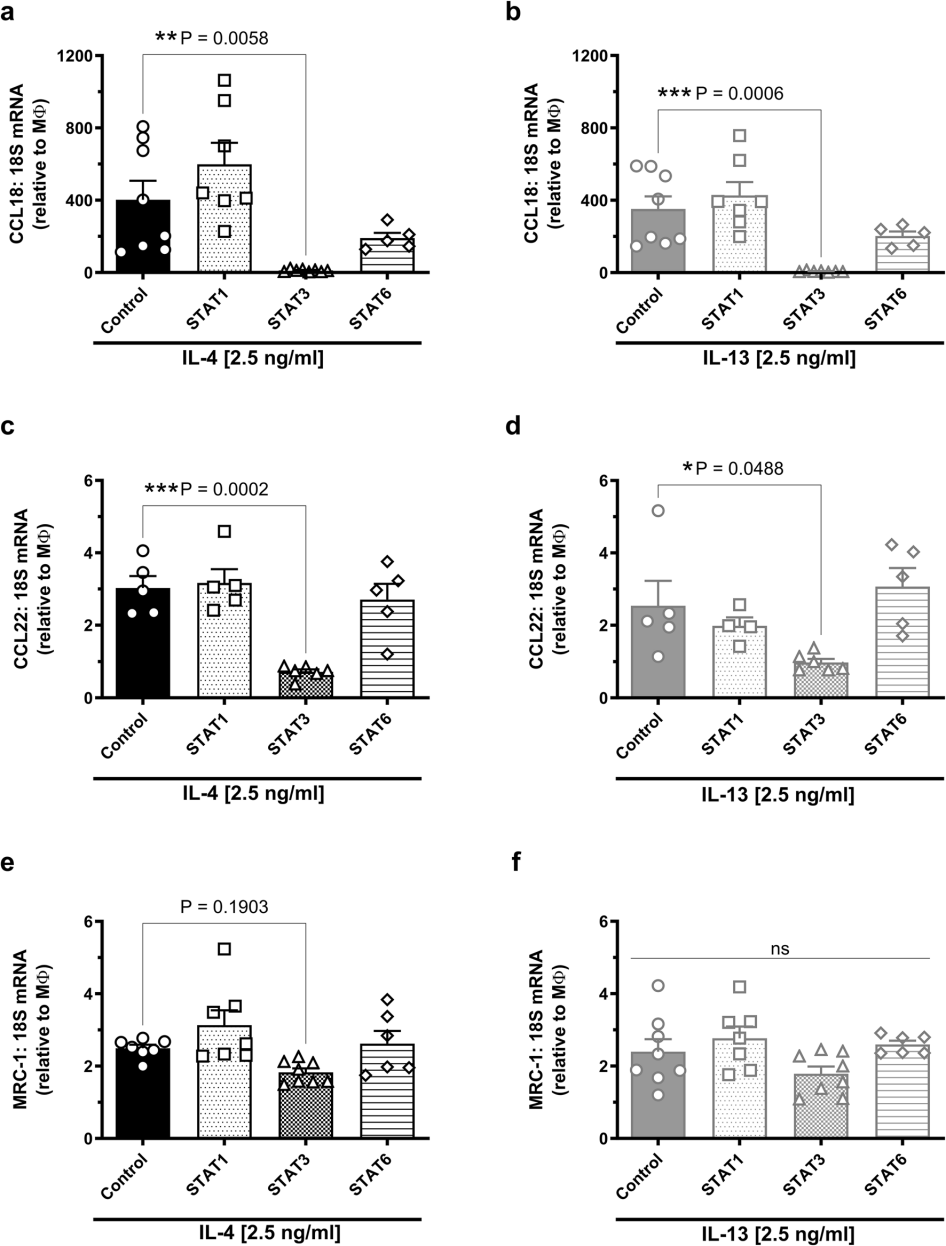
Effect of signal transduction inhibitors on the expression of M2 markers in M-CSF differentiated human primary macrophages in response to IL-4 and IL-13. M-CSF-differentiated human primary macrophages were left untreated (control) or treated with with either Fludarabine (100μM, STAT1 inhibitor), Stattic (10μM, STAT3 inhibitor), or AS1517499 (100nM, STAT6 inhibitor) in the presence of IL-4 or IL-13 (2.5 ng/ml, 24 h). Left panel: fold changes in mRNA expression of (**a**) CCL18, (**c**) CCL22 and (**e**) MRC-1 following IL-4 and inhibitors, n = 5–8. Right panel: fold-changes in mRNA expression of (**b**) CCL18, (**d**) CCL22, and (**f**) MRC-1 following treatment with IL-13 and inhibitors, n = 4–8. Signal transduction inhibitors were added 30 min prior to the addition of cytokines and maintained for the 24-h treatment period. Results presented as mean ± SEM and expressed relative to the average MΦ value (one-way ANOVA followed by Dunnett’s post hoc test).

Strikingly, CCL18 expression induced by IL-4 or IL-13 was virtually abolished (97% decrease) with STAT3 inhibition (**P = 0.006 for IL-4, ***P = 0.0006 for IL-13; Fig. 3a,b), with a trend towards a decrease with the inhibition of STAT6 (P = 0.27 for IL-4, P = 0.23 for IL-13). Similar to CCL18, IL-4- and IL-13-induced CCL22 expression was significantly decreased by 76% and 61%, respectively with STAT3 inhibition (***P = 0.0002 for IL-4, *P = 0.05 for IL-13) whereas no change was evident for STAT1 or STAT6 inhibition (Fig. 3c,d). Finally, we found that MRC-1 expression induced by IL-4 or IL-13 (both 2.5 ng/ml) was unchanged by inhibition of STAT1, 3 or 6, although a modest trend for a decrease with STAT3 inhibition was evident (P = 0.19 for IL-4, P = 0.28 for IL-13; Fig. 3e,f).

### IL-4 and IL-13 have similar effects on NADPH oxidase expression and superoxide generation

**Figure 4:**
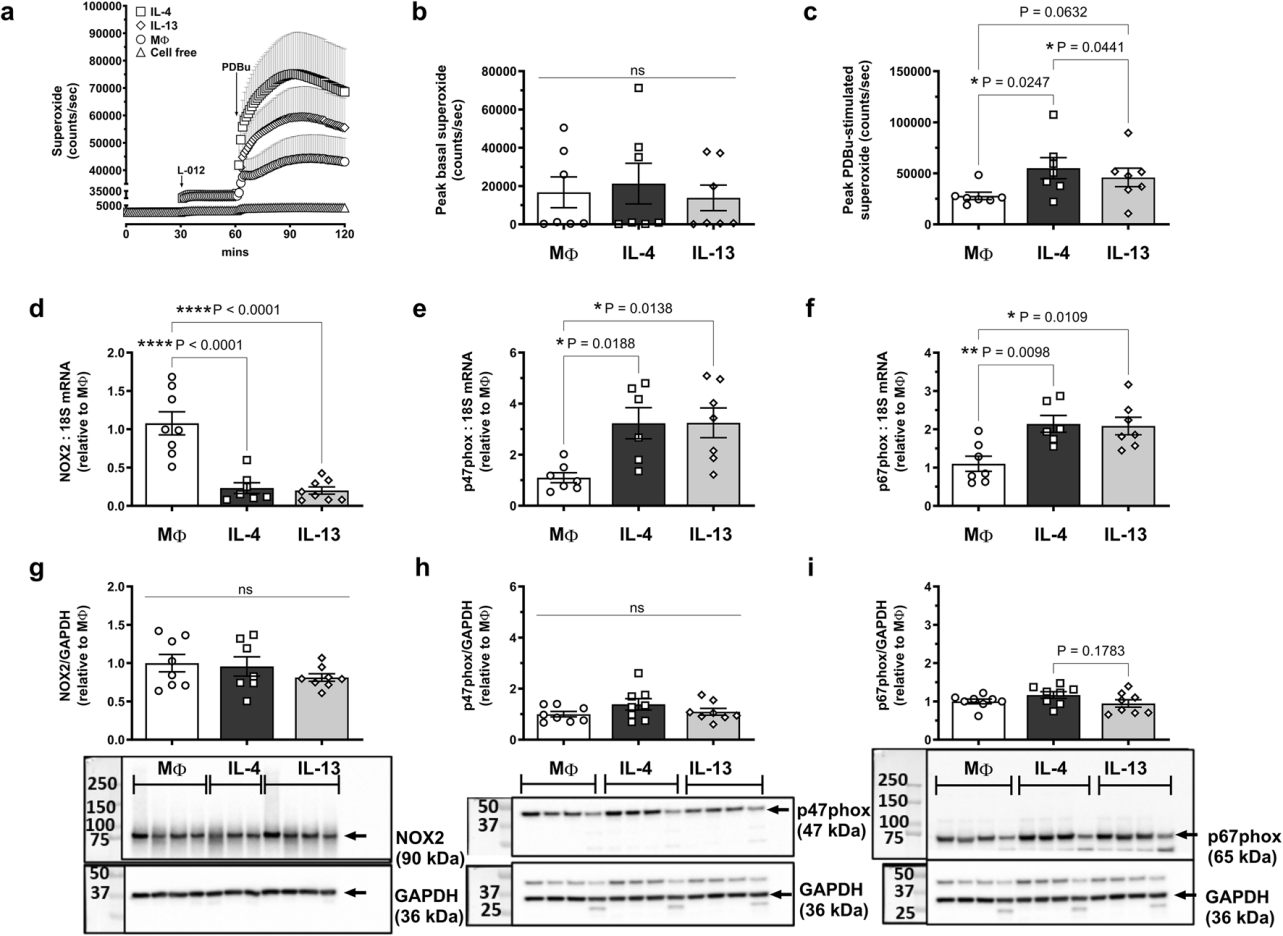
Effect of IL-4 and IL-13 on superoxide generation and NOX2 expression in M-CSF-differentiated human primary macrophages. M-CSF-differentiated human primary macrophages were left untreated (MΦ) or treated with 50 ng/ml IL-4 or IL-13 for 24 h and superoxide levels detected by L-012-enhanced chemiluminescence. (**a**) Average recording demonstrating initial background readings (1–30 min), basal superoxide as detected following L-012 (100 μM) addition (31–60 min) and PDBu (10 μM)-stimulated superoxide generation (61–120 min) measured in relative light units (RLU, counts/sec). (**b**) Peak basal (background signal subtracted) and (**c**) PDBu-stimulated (basal signal subtracted) superoxide generation. Results presented as mean ± SEM, n = 7. (Repeated measures one-way ANOVA followed by Tukey’s post hoc test). (**d–f**) mRNA and (**g–i**) protein expression of NOX2, p47phox and p67phox. Representative blots, depicting n = 3–4, are shown below each graph with GAPDH used as a loading control. Blots have been cut to probe for the protein of interest and GAPDH. Original blots are presented in Supplementary Fig. 2. Results presented as mean ± SEM and expressed relative to MΦ, n = 6–8 (one-way ANOVA followed by Tukey’s post hoc test).

We next sought to compare the ROS-generating capacities of IL-4- and IL-13-treated macrophages. Whilst IL-4 and IL-13 treatment did not alter basal superoxide levels, a significant 97% and 65% increase in PDBu-stimulated superoxide production was observed following IL-4 (*P = 0.02) and IL-13 (P = 0.06) treatment, respectively (Fig. 4a–c). However, while both cytokines elevated superoxide production compared to MΦ macrophages, the peak superoxide production induced by IL-13 was significantly less than that of IL-4, by 17% (*P = 0.04; Fig. 4a,c).

As NOX2 oxidase is the primary source of ROS in macrophages, mRNA and protein expression of its catalytic (NOX2 and p22phox), organiser (p47phox) and activator (p67phox), subunits were assessed by RT-qPCR and Western blotting. NOX2 mRNA was decreased by 80% with both cytokines (****P < 0.0001; Fig. 4d). IL-4 and IL-13 treatment induced a threefold increase in p47phox mRNA (*P = 0.02 for IL-4; *P = 0.01 for IL-13) and p67phox increased twofold (*P = 0.01; Fig. 4e,f). However, no changes in protein expression for the three subunits were detected via Western blotting (Fig. 4g–i). Expression of p22phox or NOX5 mRNA was also unchanged (Supplementary Fig. 1A,B) and neither NOX1 nor NOX4 mRNA was detected (Ct > 40).

### Increased type I IL-4 receptor expression in M1 macrophages is not associated with differential effects of IL-4 and IL-13 on M1 macrophage superoxide generation

**Figure 5:**
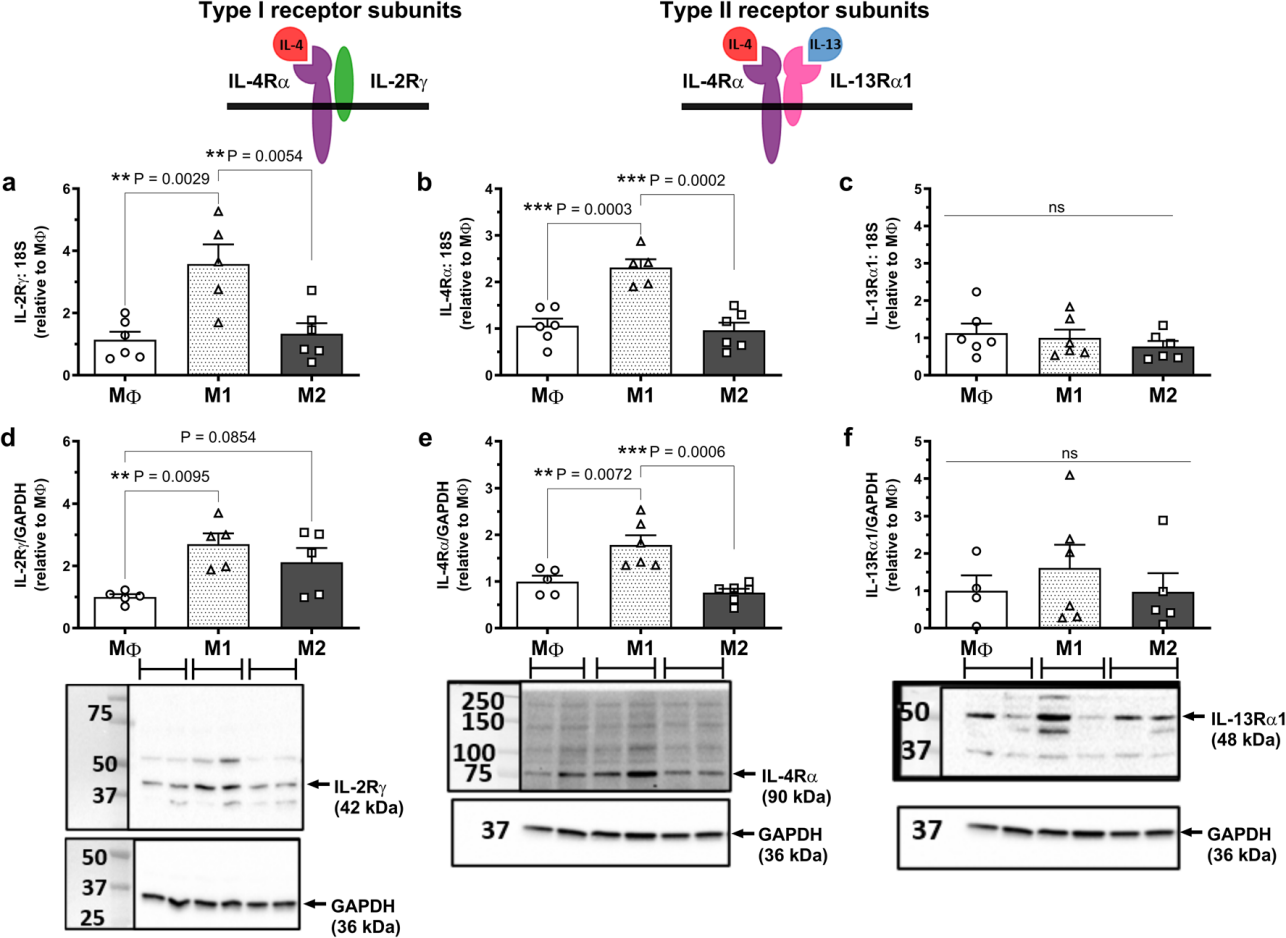
Effects of M1 polarisation on type I and II IL-4 receptor expression in human primary macrophages. M-CSF-differentiated human primary macrophages were left untreated (MΦ) or treated with 20 ng/ml IFN-γ and 100 ng/ml LPS (M1) or 25 ng/ml IL-4 (M2) for 24 h. mRNA levels of (**a**) IL-2Rγ, (**b**) IL-4Rα and (**c**) IL-13Rα1 were determined by RT-qPCR, n = 5–6. Protein levels of (**d**) IL-2Rγ, (**e**) IL-4Rα and (**f**) IL-13Rα1 were determined by western blotting, n = 4–6. Representative blots, depicting n = 2, are shown beneath each graph with GAPDH included as a loading control. Blots have been cut to probe for the protein of interest and GAPDH. Original blots are presented in Supplementary Fig. 2. The structure of the type I and II IL-4 receptor complexes are shown above data. Results presented as mean ± SEM and expressed relative to the average MΦ value (one-way ANOVA followed by Tukey’s post hoc test).

It is unknown whether macrophage subsets (M1 vs. M2) differentially express type I and type II IL-4 receptors. Interestingly, the mRNA and protein levels of both subunits of the type I receptor (IL-2Rγ and IL-4Rα) were elevated in M1, as compared to M2, or unpolarised macrophages. For IL-2Rγ, mRNA expression was increased 3.6-fold (**P = 0.003 vs. MΦ; **P = 0.005 vs. M2), and protein expression upregulated by 2.7-fold (**P = 0.095 vs. MΦ; Fig. 5a,d). An mRNA elevation of 2.3-fold (***P = 0.0003 vs. MΦ; ***P = 0.0002 vs. M2) and a 1.8-fold increase in protein (**P = 0.007 vs. MΦ; ***P = 0.0006 vs. M2) was evident for IL-4Rα expression (Fig. 5b,e). However, the IL-13-specific subunit, IL-13Rα1 was unchanged at the mRNA and protein level between all groups (Fig. 5c,f). Due to this bias in expression toward the type I receptor, which may only be targeted by IL-4, we hypothesised that IL-4 may have more potent effects on M1 macrophages as opposed to IL-13 and compared the effects of IL-4 and IL-13 on M1 macrophage superoxide generation.

**Figure 6:**
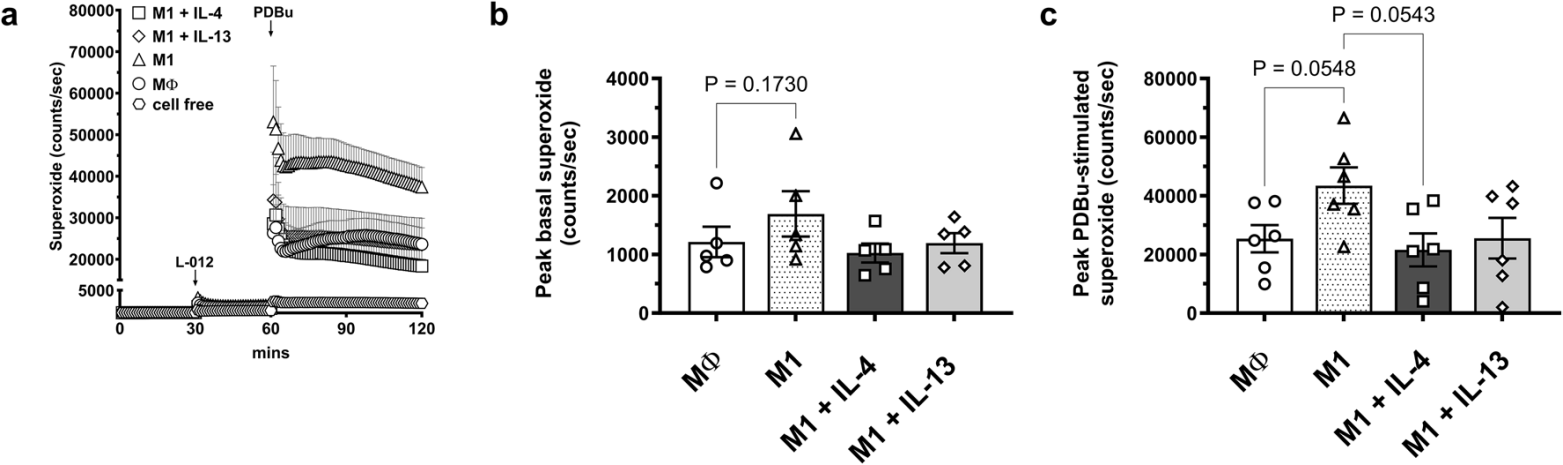
Effects of IL-4 and IL-13 on superoxide generation in M1-polarised human primary macrophages. M-CSF-differentiated human primary macrophages were left untreated (MΦ) or polarised to the M1 phenotype for 18 h prior to the addition of IL-4 or IL-13 (50 ng/ml) for a further 6 h. Subsequently, superoxide levels were measured by L-012-enhanced chemiluminescence. (**a**) Average recording demonstrating initial background readings (1–30 min), basal superoxide as detected following L-012 (100 μM) addition (31–60 min) and PDBu (10 μM)-stimulated superoxide generation (61–120 min) measured in relative light units (RLU, counts/s). (**b**) Peak basal (background subtracted) and (**c**) PDBu-stimulated (basal subtracted) superoxide generation. Results presented as mean ± SEM, n = 5 (repeated measures one-way ANOVA followed by Sidak’s post hoc test).

Basal superoxide generation was unchanged between all groups (Fig. 6a,b), however, M1 macrophage polarisation resulted in a significant increase in PDBu-stimulated superoxide production compared to MΦ (P = 0.055; Fig. 6a,c). Although IL-4 and IL-13 alone promoted macrophage superoxide generation (Fig. 4), addition of IL-4 for the final 6 h of the 24 h M1 polarisation treatment attenuated PDBu-stimulated superoxide production in M1 macrophages by 50% (P = 0.054). A similar non-significant trend (P = 0.31 for M1 + IL-13 vs M1) was observed for IL-13 and both cytokines prevented the observed increase in M1 vs MΦ macrophages (Fig. 6a,c).

## Discussion

The closely related Th2 cytokines, IL-4 and IL-13, can act through the shared type II IL-4 receptor to contribute to allergic inflammation and fibrotic diseases^2^. Interestingly, opposing roles for these cytokines in the development of cardiovascular disease is evident with pathological and protective properties being reported in vivo. Given that macrophages are one of just a few cell types reported to express both the type I and type II IL-4 receptors^20^, we investigated whether these differential roles may be through distinct effects on macrophage function. Despite confirming the expression of both type I and type II IL-4 receptors in human primary macrophages, we found that IL-4 and IL-13 exhibited equivalent potency for the induction of M2 marker expression and only subtle differences in the generation of ROS.

Whilst expression and activity of both the type I and type II IL-4 receptors has been reported in human peripheral blood monocytes^18,21,33^, there has been limited assessment of their expression following differentiation to macrophages. Here we show for the first time that human macrophages express both type I and II IL-4 receptor mRNA at equivalent levels, but lack expression of the decoy receptor, IL-13Rα2, findings which are consistent with those in human peripheral blood monocytes and mouse bone marrow derived macrophages^20,34^. We were also able to demonstrate protein expression of each IL-4 receptor subunit via Western blotting, suggesting that both receptors are present on human primary macrophages. Interestingly, Western blotting revealed a modest increase in both IL-4Rα and IL-13Rα1 following IL-4, but not IL-13 treatment. These subunits form the type II IL-4 receptor thus, this finding may suggest that M2 macrophages initially polarised by IL-4 may in turn be more responsive to both IL-4 and IL-13 signalling via this receptor subtype.

Having confirmed IL-4 receptor expression, we next sought to compare the ability of IL-4 and IL-13 to promote M2 macrophage polarisation. Previous investigations have shown enhanced potency of IL-4, as compared to IL-13, to promote M2 marker expression (Arg1) in murine macrophages, attributed to signalling through the type I receptor^20,34^. Another study however, in agreeance with our report, found no differences between IL-4 and IL-13 as an M2 stimulus in human primary macrophages in terms of phenotypic profile by flow cytometry and gene expression analysis^35^. Tarique et al.^35^, further noted that IL-4 and IL-13 promoted the expression of three M2 markers (including CCL18), a finding similar to ours. Furthermore, they found that IL-13-polarised macrophages release significantly greater amounts of IL-13 compared to those treated with IL-4, but both release equivalent quantities of CCL18. This suggests that IL-13, acting on the type II receptor may initiate a feedback loop to produce more IL-13 to polarise additional macrophages to a potentially beneficial IL-13-dominant state. Unfortunately, the authors did not study IL-4 release following macrophage polarisation with the two cytokines, data which would be of interest to obtain in future studies.

Building from these previous studies we demonstrated equivalent potency and efficacy for IL-4 and IL-13 to upregulate established M2 markers (MRC-1^36^, CCL18^35^ and CCL22^37^). Given the type II IL-4 receptor is common to both cytokines, our observations suggest that signalling to induce M2 polarisation in human primary macrophages is predominantly via this receptor. Since M-CSF-differentiated human primary macrophages are the most clinically relevant cell type for such studies, our data may inform on how these receptors may be selectively exploited for the development of therapeutics. However, as we also demonstrated type I IL-4 receptor expression in human primary macrophages, future investigation should selectively inhibit either the type I (IL-2Rγ) or type II (IL-13Rα1) specific subunits to delineate the potential contribution of both receptor subtypes to IL-4 signalling.

IL-4 and IL-13 can activate a different suite of signalling cascades depending on the receptor type and subunits they target. To gain further insight into the modulation of macrophage phenotype by the Th2 cytokines, we examined the contribution of distinct signalling pathways to the induction of M2 markers by IL-4 and IL-13, specifically STAT1, 3 and 6. Type I receptor signalling is associated with STAT3 and STAT6 transduction whereas the type II receptor has been reported to signal through STAT1, STAT3 and STAT6. Importantly, these transduction pathways are more specifically associated with particular subunits and cytokines acting on these receptors; IL-4Rα binding with IL-4 or IL-13 is linked to STAT3 and STAT6^2,38^, IL-13Rα1 targeted by either IL-4 or IL-13 with STAT6, but only IL-13 (and not IL-4) binding to IL-13Rα1 is associated with STAT1^21^.

In regard to the signal transduction pathways associated with IL-4 and IL-13, in this study it was observed that the STAT3 inhibitor, Stattic, almost completely abolished the IL-4- and IL-13-induced increases in mRNA expression of the M2 marker, CCL18, significantly lowered CCL22 expression induced by both cytokines and trended towards blocking MRC-1 expression; observations which have not been reported previously. Both IL-4 and IL-13, acting via the IL-4Rα subunit common to the type I and II receptors, have been linked to STAT3 signalling^2,21,38^, albeit not in direct relation to induction of an M2 phenotype. Thus, it is plausible that inhibition of this particular pathway could affect responses to both cytokines and be a major contributor to the similarity of the effects observed between the two in this study.

STAT6 signalling is associated with both the type I and type II receptor and previous studies have strongly linked the M2 phenotype (as characterised by MRC-1 expression) to a STAT6-dependent signalling pathway^22,23,39^. Thus, while we anticipated that the effects of IL-4 and IL-13 on macrophage phenotype in the present study would likely involve STAT6, our data showing no inhibitory effect of the STAT6 inhibitor, AS1517499, suggested otherwise. Before we can definitively exclude a role for STAT6, it will be necessary to interrogate this pathway further with different methods of STAT6 inhibition such as siRNA. It is also important to recognise that many additional factors act in concert to promote M2 polarisation in macrophages^39^, particularly when considering an in vitro vs. in vivo environment. In addition, STAT3 and STAT6 may both be engaged in the response to IL-4 and IL-13, such that inhibition of both signalling pathways is required to observe an impact on MRC-1 expression.

Although both IL-4 and IL-13 appeared to promote M2 polarisation via the type II IL-4 receptor, this does not preclude an ability of IL-4 to also target the type I IL-4 receptor and differentially modulate potentially detrimental downstream mediators, such as ROS. NOX2 oxidase is the major source of ROS in macrophages^40^ and although more commonly associated with M1 macrophage function, its activity is also observed in alternatively activated macrophages^41^. With this in mind, we wished to determine whether NOX2-derived ROS may be more prevalent in M2 macrophages polarised with IL-4, as compared to IL-13 and thus, be involved in the potentially opposing roles of these cytokines in cardiovascular disease. We found that PDBu-stimulated superoxide production was increased following IL-4 treatment, as compared to untreated macrophages, but this effect was less pronounced with IL-13. It is important to point out that these differences were rather small and there was a degree of variability. Interestingly, these increases in superoxide coincided with increases in p47phox and p67phox at the level of mRNA but this was not associated with a change in protein expression of either NOX2, p47phox or p67phox. As such, the mechanisms underlying the ability of IL-4 to increase superoxide to a slightly greater extent than IL-13 are unclear, but may be related to additional signalling associated with IL-4 targeting the type I receptor, a concept which awaits clarification in future studies.

Another potential point of difference between IL-4 and IL-13 is in their actions on M1 macrophages. Interestingly, we found that M1 macrophages have increased expression of both the IL-4Rα and IL-2Rγ subunits of the type I receptor, but not IL-13Rα1, suggesting the potential for additional, or more potent, effects of IL-4, as compared with IL-13, on these cells. Indeed, LPS-stimulated increases in IL-2Rγ^42^ and IL-4Rα^43^ have previously been reported on monocytes and microglia, respectively. Importantly however, this is the first demonstration of enhanced type I IL-4 receptor expression on M1 macrophages, which could have implications in a range of inflammatory diseases. Whilst the reasons underlying the greater expression of the IL-4 receptor on M1 as compared to M2 macrophages is unknown, the lack of modulation of the IL-13Rα1 subunit (required for IL-13 activity) following M1 polarisation suggests that IL-4 may modulate M1 function to a greater extent than IL-13.

Considering our observation that IL-4 increased macrophage ROS generation whereas IL-13 did so to a lesser extent, we proposed that IL-4 may further enhance ROS production from M1 macrophages. As anticipated, M1 polarised macrophages exhibited increased superoxide production compared to MΦ. However, neither IL-4 nor IL-13 further augmented superoxide generation, rather there was a trend for both cytokines to attenuate superoxide generation in M1 macrophages. As such, these findings suggest that Th2 cytokines, may serve to limit the damaging effects of M1 macrophage-derived ROS in cardiovascular disease, a concept which requires further interrogation. Importantly, these results do not preclude differential actions of IL-4 and IL-13 on other M1 functions not investigated in this study, such as pro-inflammatory cytokine release.

It is important to note that, in contrast to our controlled in vitro experiments, the vascular microenvironment and the in vivo environment per se are highly complex. Whilst we didn’t observe any major differences in function between IL-4- and IL-13-stimulated macrophages in our experiments, the potential for interaction with other stimuli or targets remain to be elucidated. Indeed, in vivo studies have failed to provide more clarity on the subject and have demonstrated somewhat conflicting results for these Th2 cytokines in animal models of cardiovascular disease. Some studies exist suggesting that IL-4 is indeed detrimental, and IL-13 is protective^8,9,14,17,44–46^, whilst others suggest IL-4 and/or IL-13 are protective^11,13,47,48^. Furthermore, despite its lack of constitutive expression on macrophages, a role for the IL-13Rα2 decoy receptor cannot be excluded in an in vivo setting. For example, in an environment in which both Th1 and Th2 cytokines are present, IL-13Rα2 expression could be induced^49,50^. Cell surface IL-13Rα2 binds IL-13 with extremely high affinity^19^ and there is evidence that, in addition to its reported decoy function, the receptor can signal in response to IL-13 to have pro-fibrotic effects^50,51^. Hence some of the protective actions of IL-13 in cardiovascular disease could be mediated via the IL-13Rα2 receptor, leading to increased collagen deposition and plaque stability, or reparative connective tissue formation following MI.

The similar actions of IL-4 and IL-13 on M1 and M2 macrophages suggests that the opposing roles of these cytokines in cardiovascular disease may be via effects on other cell types. Specifically, IL-13 is more strongly implicated in fibrosis and transforming growth factor-β1 (TGF-β1) signalling through its actions on non-immune cells^52^. By contrast IL-4 may favour inflammatory responses as it has been shown to promote oxidative stress and adhesion molecule expression in endothelial cells^28^. Alternatively, beneficial and detrimental roles of the two cytokines may differ depending on the particular disease state or may have arisen due to differences in the experimental design of the various investigations. Thus, to date, no study has directly compared the effects of single cytokine IL-13 or IL-4 deficiency, or exogenous treatment, in the same cohort of diseased animals. Such an approach, in addition to comparing the effects of IL-4 and IL-13 on vascular cells, is required to provide definitive evidence for differential effects of these cytokines in a whole-body system.

## Conclusions

In conclusion, we have confirmed the expression of both the type I and type II IL-4 receptors on human primary macrophages but observed no substantial differences in the effects of IL-4 and IL-13 on macrophage function in the context of polarisation, signalling, or superoxide generation. A modest ability of IL-4 to augment macrophage superoxide generation as compared to IL-13 is unlikely to have a major physiological impact that would explain opposing roles in cardiovascular disease such as atherosclerosis, hypertension, or MI. Taken together, our findings suggest that the potentially opposing roles of IL-4 and IL-13 are not through direct actions on macrophages, rather they may reflect differential modulation of other cell types, such as endothelial or vascular smooth muscle cells. Future studies should focus on a direct comparison of IL-4 and IL-13 as well as their cytokine-specific receptor subunits (i.e., IL-2Rγ and IL-13Rα1, respectively) in the context of cardiovascular disease in vivo to validate the therapeutic potential of targeting one cytokine over the other.

### Supplementary Information

Supplementary Information.

Supplementary Figures.

## Data Availability

Gene expression data is available in the Supplementary Dataset File.
