# Supplementary material for: IL-4 and IL-13 induce equivalent expression of traditional M2 markers and modulation of reactive oxygen species in human macrophages
Source: Sci Rep. 2023 Nov 10;13:19589. doi: 10.1038/s41598-023-46237-2 (PMC10638413; doi:10.1038/s41598-023-46237-2)

Equivalent macrophage responses to IL-4 and IL-13: impact on phenotype and reactive oxygen species generation

Tara E. Scott[1†], Caitlin V. Lewis[1†], Mingyu Zhu[1], Chao Wang[1], Chrishan S. Samuel[1], Grant R. Drummond[2], Barbara K. Kemp-Harper[1*]

[1]Cardiovascular Disease Program, Biomedicine Discovery Institute & Department of Pharmacology, Monash University, VIC, Australia

[2]Department of Physiology, Anatomy & Microbiology, School of Life Sciences, La Trobe University, VIC, Australia

[†] these authors have contributed equally to this work

Supplementary Data

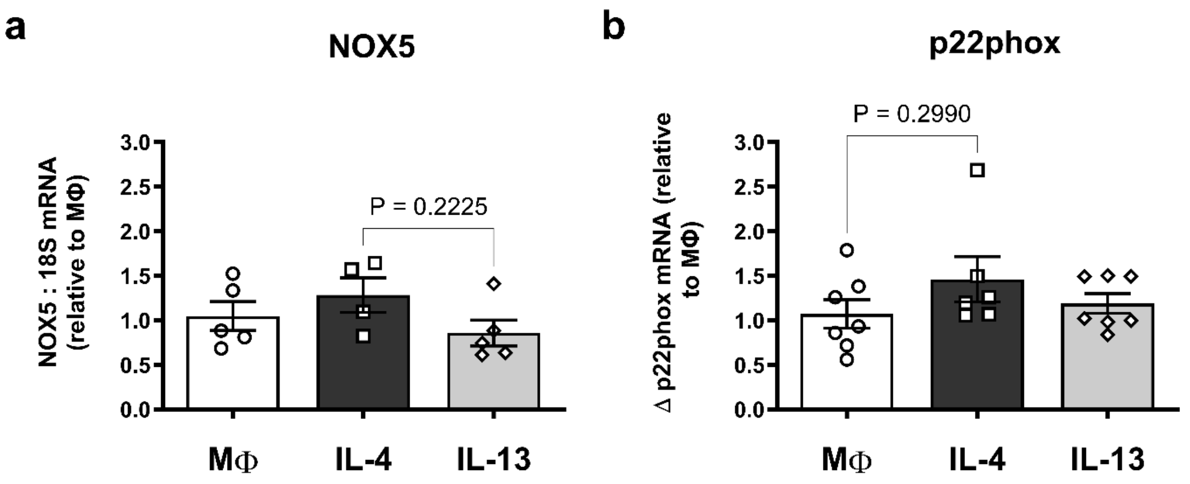

Supplementary Figure 1. Effect of IL-4 and IL-13 on mRNA expression of additional NOX isoforms and subunits in human primary macrophages. M-CSF-differentiated human primary macrophages were left untreated (MΦ) or treated with 50 ng/ml IL-4 or IL-13 for 24h. mRNA levels of p22phox **a)**, NOX5 **b)**, NOX1 and NOX4 were determined by RT-PCR, n=4-7. NOX1 and NOX4 isoforms were not detected in any treatment group (Ct value = >40). Results presented as mean ± SEM and expressed relative to the average MΦ value. One-way ANOVA followed by Tukey's post hoc test.

a

b

c

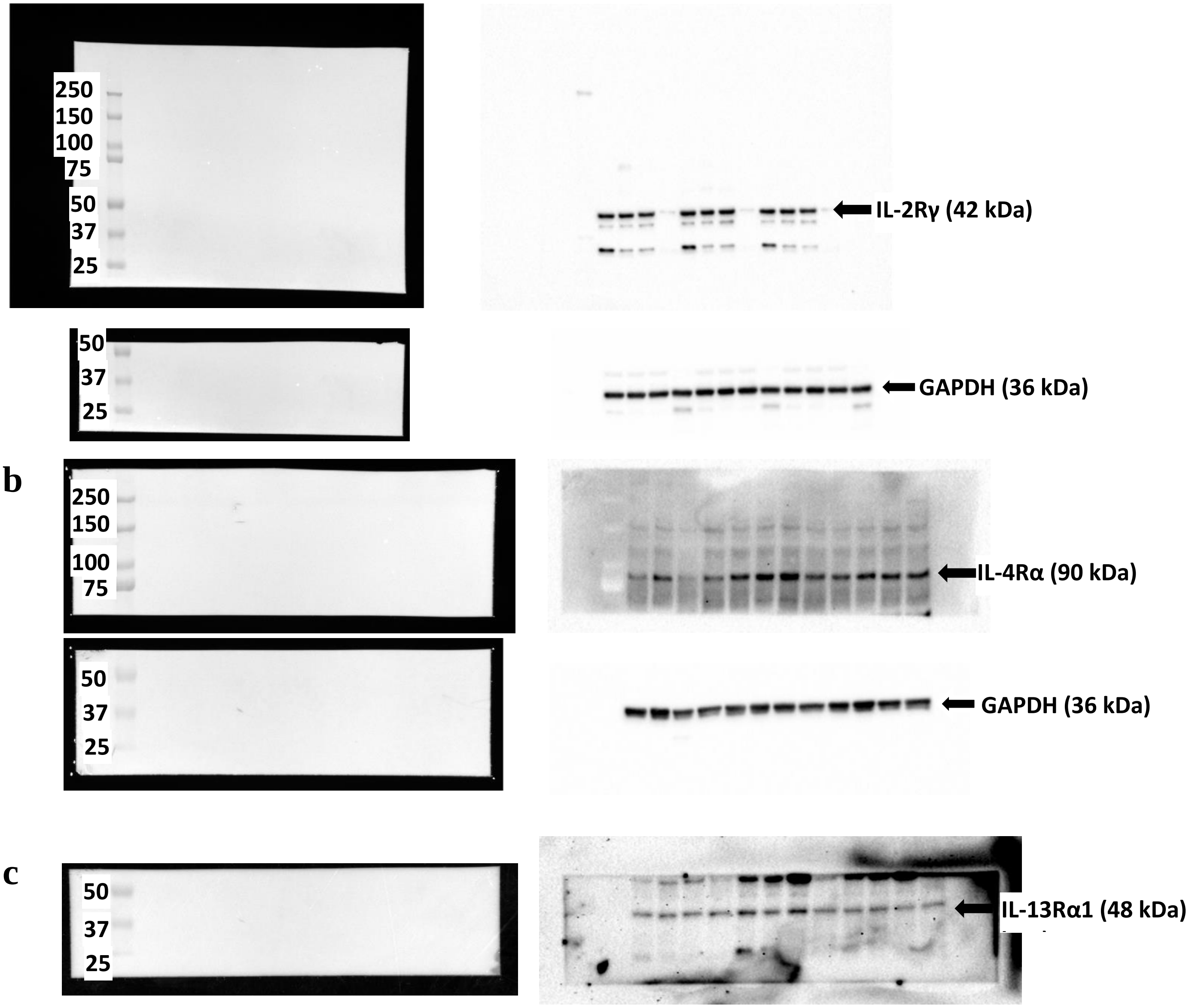

-same GAPDH as for b

Supplementary Figure 2: Full length blots, including molecular size markers (kDa; LHS-membrane image aligned with chemiluminescence image on RHS), for protein expression data used for representative images shown in **a)** Figure 1d (Mθ-lanes 1-3, IL-4- lanes 5-7, IL-13- lanes 9-11; lanes 4, 8 and 12 were from the same batch of PBMCs that had very low receptor expression and were excluded as outliers), **b)** Figure 1e (Mθ-lanes 1-4, IL-4- lanes 5-8, IL-13- lanes 9-12), and **c)** Figure 1f (Mθ-lanes 1-4, IL-4- lanes 5-8, IL-13- lanes 9-12). Blots were first probed for IL-2Rγ, stripped, cut above 50 kDa and then re-probed p67phox (upper; Supplementary Figure 3c) and p47phox (lower; Supplementary Figure 3b), then GAPDH (lower). Replicate blots were first cut above 50 kDa, probed for NOX2 (upper; Supplementary Figure 3a) and IL-13Rα1 (lower) then stripped and re-probed for IL-4Rα (upper) and GAPDH (lower).

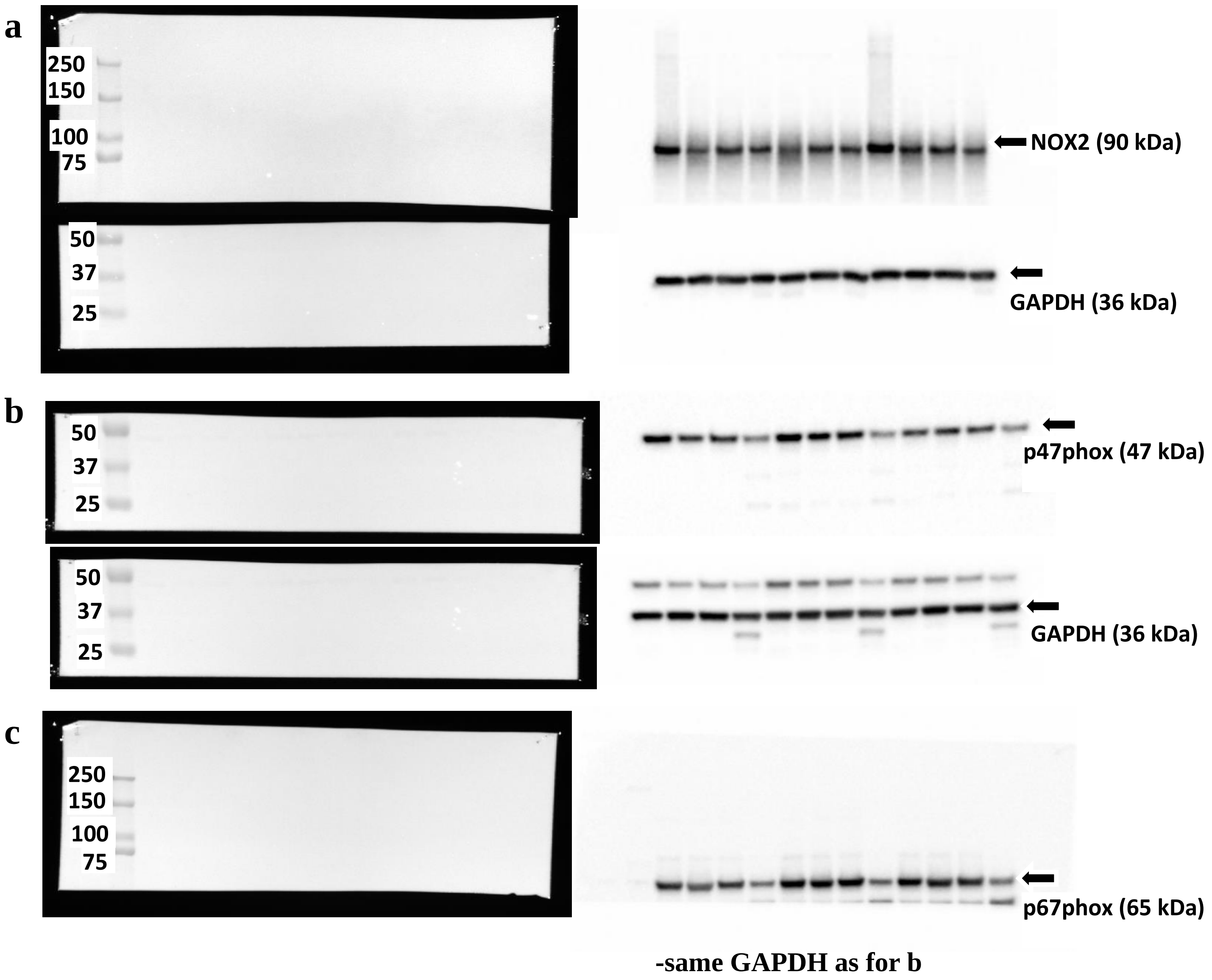

Supplementary Figure 3: Full length blots, including molecular size markers (kDa; LHS- membrane image aligned with chemiluminescence image on RHS), for protein expression data used for representative images shown in **a)** Figure 4g (Mθ-lanes 1-4, IL-4- lanes 5-7, IL-13- lanes 8-11) , **b)** Figure 4h (b: Mθ-lanes 1-4, IL-4- lanes 5-8, IL-13- lanes 9-12), and **c)** Figure 4i (c: Mθ-lanes 1-4, IL-4- lanes 5-8, IL-13- lanes 9-12) in the manuscript. Blots were first probed for IL-2Rγ (Supplementary 2a), stripped, cut above 50 kDa and then re-probed p67phox and p47phox (lower; Supplementary Figure 3b), then GAPDH (lower). Replicate blots were first cut above 50 kDa, probed for NOX2 and IL-13Rα1 (lower; Supplementary Figure 2c) then stripped and re-probed for IL-4Rα (upper; Supplementary Figure 2b) and GAPDH (lower).

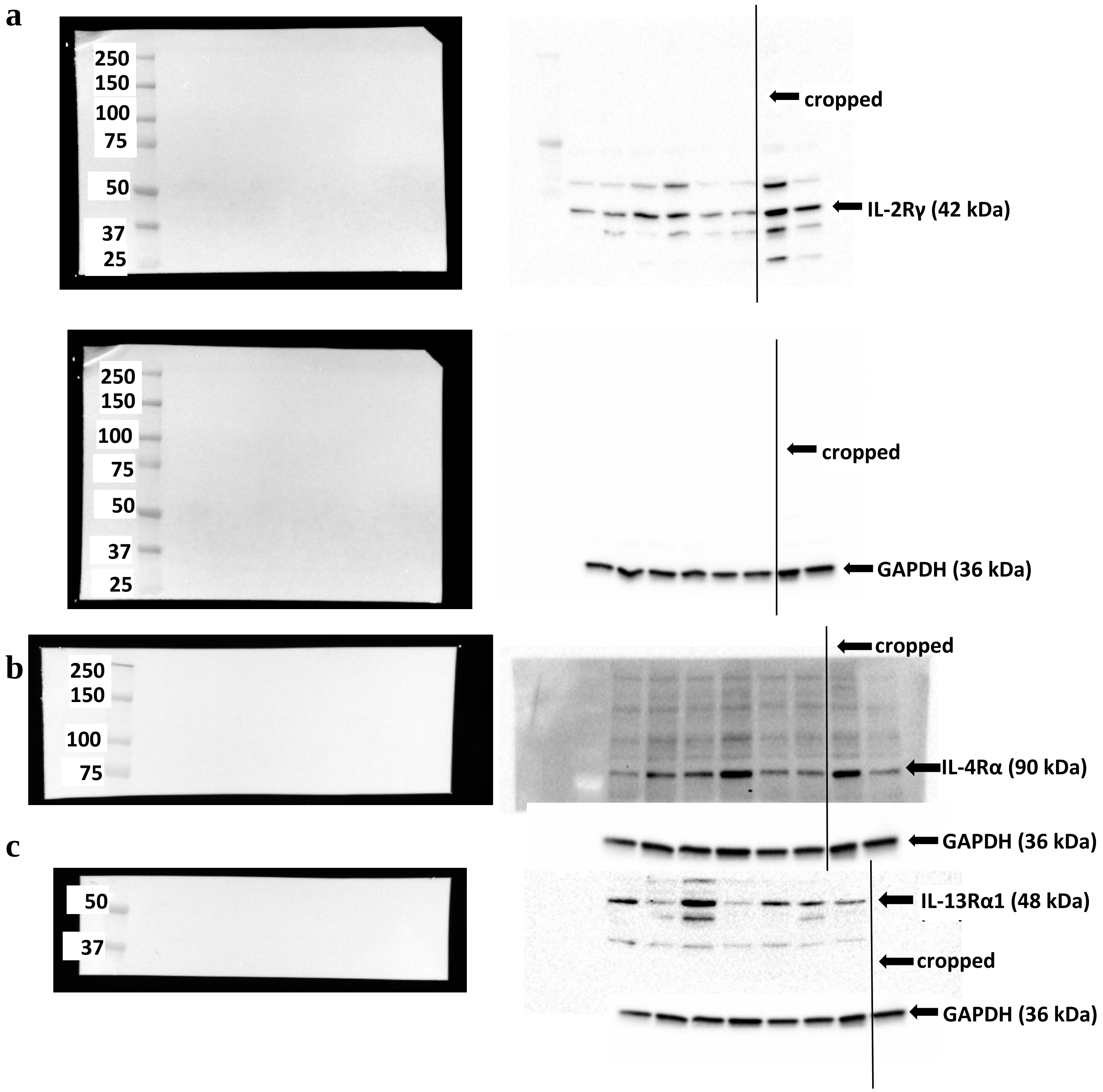

Supplementary Figure 4: Full length blots, including molecular size markers (kDa; LHS-membrane image aligned with chemiluminescence image on RHS), for protein expression data used for representative images shown in **a)** Figure 5d, **b)**, Figure 5e, and **c)** Figure 5f in the manuscript. Blots were first probed for IL-2Rγ, stripped, cut between 75 and 50 kDa, then re-probed for IL-4Rα (upper) and GAPDH (lower). Replicate blots were first probed for IL-13Rα1 then stripped and re-probed for GAPDH. All blots: Mθ-lanes 1-2, M1- lanes 3-4, M2- lanes 5-6, additional samples not used in analysis were loaded in lanes 7-8 and cropped out of representative images as indicated by vertical black lines.

Additional WBs used in data analysis:

1e

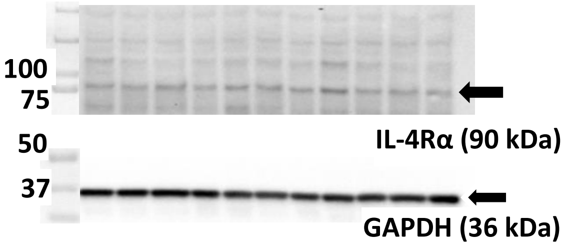

1f

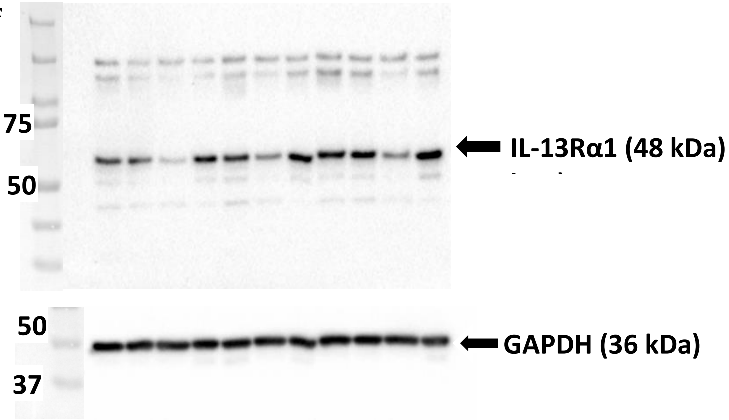

4g

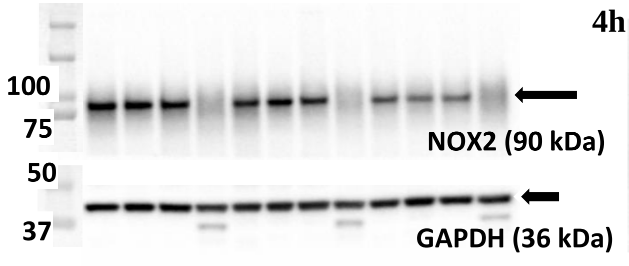

4h

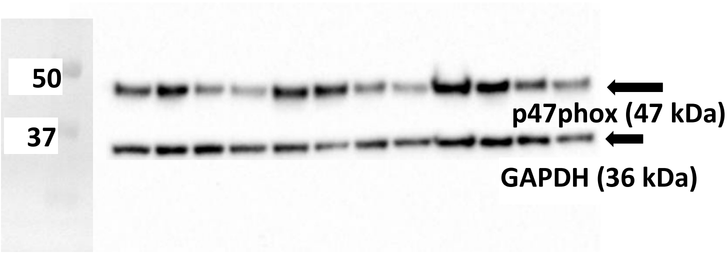

4i

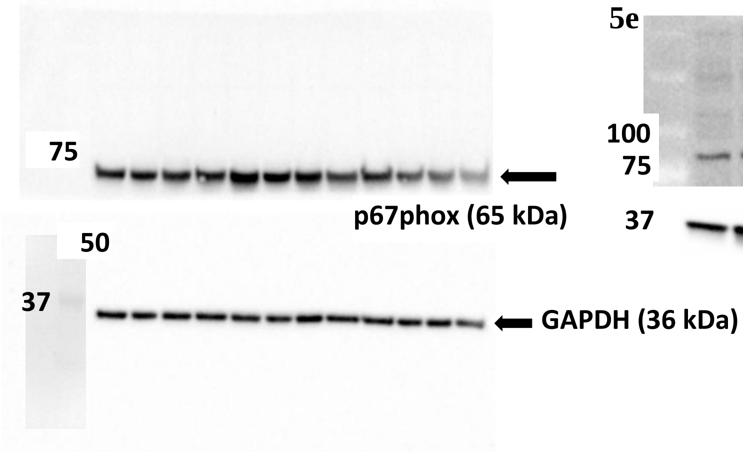

5e

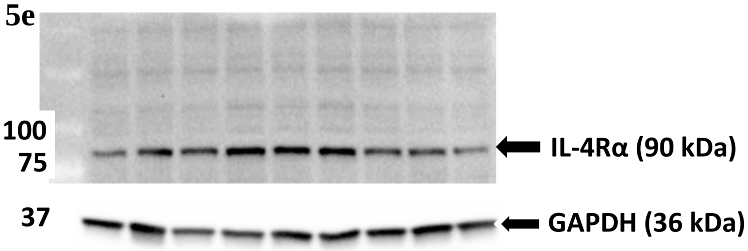

Supplement: Supplementary file 2 — Supplementary Figures. [file 41598_2023_46237_MOESM2_ESM.pdf]
